# Advanced Materials for Clothing and Textile Engineering

**DOI:** 10.3390/ma16093407

**Published:** 2023-04-27

**Authors:** Snježana Firšt Rogale, Dubravko Rogale

**Affiliations:** University of Zagreb Faculty of Textile Technology, 10000 Zagreb, Croatia; dubravko.rogale@ttf.unizg.hr

The main objective of this Special Issue is to showcase outstanding papers presenting advanced materials for clothing and textile engineering. Advanced materials have improved the performance of conventional and protective clothing and accelerated the development of e-clothing and smart clothing. The main directions of progress have become visible in recent years and are reflected in the research on and development of new materials and the improvement of their properties. Special treatment methods for textile materials are being developed to improve their use properties as well as textile care. This is of great importance for textile and clothing engineering.

This Special Issue brings together several outstanding articles on a wide range of topics, including the impact of textile production and waste on the environment, the properties of yarns and textile materials, and the wearing comfort of textiles and clothing.

Environmental pollution is a major problem and a very important research topic. Microplastics have become one of the major environmental hazards. Thus, it is imperative to investigate the main potential sources of microplastic pollution on the environment. Šaravanja et al. gave an overview of washable polyester materials. They stated that the main goals are to produce polyester with optimal structural parameters and to switch to recycled production as much as possible, including the use of the most appropriate agents that protect the structure of polymer while preventing the release of microplastics during washing. Another goal is to optimize the washing and drying processes of synthetic materials [1].

The importance of cleaning is evident in the use of textile materials for protection against electromagnetic radiation, which is suitable for composite structures of garments and for technical and interior applications. The authors evaluated the stability of the materials’ shielding properties against EM radiation after the application of apolar and polar solvents, in synergy with a cyclic process involving the parameters of wet and dry cleaning [2].

The consumption of eco-friendly fibers with a decrease in synthetic fibers, which can reduce pollution generated by the textile industry, was studied in [3].

Nanotechnology, more specifically electrospun nanofibers, has been identified as a potential solution for developing efficient filtration systems. In one study, the electrospinning technique for producing polyamide nanofibers was optimized by varying several parameters, such as polymer concentration, flow rate, and needle diameter. The optimized polyamide nanofibers were combined with polypropylene and polyester microfibers to construct a multilayer and multiscale system with increased filtration efficiency [4].

The comfort of wearing textiles and clothing is the focus of a few papers. Čubrić et al. examined the mechanical properties of polyurethane materials used to construct inflated thermal-expanding insulation chambers that serve as adaptive thermal layers in intelligent clothing, as well as their efficiency in providing thermal protection [5].

An increasing number of companies are developing advanced high-tech sportswear, often with high compression, for professional athletes. Analysis of the effects of sportswear compression on the physiological comfort of athletes is a very important topic [6].

The moisture vapor permeability and thermal wear comfort of bamboo and Tencel as environmentally friendly fibers, together with fiber core and polypropylene, nylon, and Coolmax^®^ as core filaments, were investigated in [3].

The flammability of protective clothing systems for firefighters is increasingly being investigated. Hursa Šajatović et al. presented studies on a clothing system for protection against heat and flames using a fire manikin and systematically analyzed the damage caused after testing [7].

The effects of washing parameters on the thermal properties and appearance of Proban^®^ flame-retardant material were investigated in one study. This research focused on the effects of washing conditions on the effluent composition and durability of the flame-retardant material’s properties and the appearance of Proban^®^ cotton fabrics [8].

Knezić et al. analyzed the effect of the number of electrically conductive yarns woven into a fabric on the values of electrical resistance. They observed how the direction of action of the elongation force affects the change in electrical resistance of the electrically conductive fabric, taking into account the position of the interwoven electrically conductive yarns [9].

Ielina et al. studied the geometric parameters of a knitted loop. In this paper, a mathematical description of the coordinates of the characteristic points of the loop and an algorithm for calculating the coordinates of the control vertices of the second-order spline, which determine the configuration of the yarn axes in the loop, are presented [10].

Knitted fabrics are subjected to dynamic loading due to body movements during their use. The influence of elastane content on the elastic properties of knitted fabrics under dynamic loading was investigated in [11].

Stockings must be made of high-quality material and provide comfort to wearers. Stockings that were knitted in a plain single jersey pattern and made with the highest percentage of ring, rotor, and air-spun modal or micromodal yarns of the same linear density in full plating with various textured polyamide 6.6 yarns were investigated in [12].

A very interesting area of research is the new approach to the creation process in fashion design that results from the use of thermal camouflage in the design of clothing. In one study, the main variation factors of thermal images were determined by analyzing their color behavior in a daytime and nighttime outdoor environment in the presence and absence of a dressed human body through the use of a thermal imaging camera [13].

Drape is one of the most important characteristics associated with the quality and attractiveness of a garment. Memon et al. investigated how bending stiffness and thickness affect the drapeability of garment leathers. This study also has practical implications as it can help practitioners better understand these elements and select appropriate materials for garment companies and customers [14].

Petrak et al. presented an analysis of the parameters of a polygonal computer model that affect fabric drape simulations. The fabric drape simulations were performed using the 2D/3D CAD system for computer clothing design on a disk model, which corresponded with the real tests using a drape tester; the aim was to perform a correlation analysis between the values of the drape parameters of the simulated fabrics and the realistically measured values for fabrics [15].

Virtual prototyping is a technique in the apparel development process that involves the use of computer-aided design to develop apparel and their virtual prototypes. Virtual simulations of trousers and real-trouser prototypes were compared to investigate their fit and comfort on scanned and kinematic 3D body models, as well as on a real body. By changing the body posture, the real and virtual body circumferences were changed, which affected the fit and comfort of the virtual and real trousers [16].

Bogović et al. described a study on the use of 3D printed knee protectors intended for wheelchair users. The construction of clothing and the 3D modeling of the elements integrated into the garment were interdependent, and the design solutions were found to provide adequate and reusable garments, especially for sensitive target groups such as people with disabilities [17].

The properties of flame-retardant fabrics and the possibility of their finishing in the processes of dyeing and printing were studied. The possibility of reactive printability on protective flame-resistant fabrics varying in the composition of weft threads and weave was studied, and the washfastness of the printed samples was analyzed [18].

Kumpikaitė et al. investigated the distribution of crimp in new jacquard fabric structures combining single- and double-ply weaves and fabric width to provide a method for predicting crimp [19].

The pilling resistance of fashion fabrics is a fundamentally important and common problem when wearing clothes. The aim of one study published in this Special Issue was to evaluate the pilling behavior of linen/silk fabrics with different mechanical and chemical finishes and to determine the influences of the raw materials and the specifics of dyeing and digital printing with different dyes [20].

In another study, the first descriptive bibliometric analysis to study the most influential journals, institutions, and countries in the field of artificial intelligence in the textile industry was conducted. The analysis covered all major areas of artificial intelligence, including data mining and machine learning [21].

The articles published in this Special Issue show that the field of textile and clothing engineering is experiencing extraordinary development dynamics. We are pleased that the Editorial Office of *Materials* has recognized this trend and made possible the publication of this Special Issue. The published articles highlight new trends and address many important issues in the development of advanced materials for textile and clothing engineering. 
